# Supplementary material for: Smooth Interpolating Curves with Local Control and Monotone Alternating Curvature
Source: Comput Graph Forum. 2022 Oct 6;41(5):25–38. doi: 10.1111/cgf.14600 (PMC9827861; doi:10.1111/cgf.14600)
Supplement: Supplementary file 1 — Supplement Material [file CGF-41-25-s001.zip › Local-Smooth-Interpolating-MonoCurvature/extern/clothoids/submodules/Utils/docs/api-cpp/program_listing_file_Console.cc.html]

Program Listing for File Console.cc — Utils v1.2

### Navigation

- index
- toc
- Utils »
- Program Listing for File Console.cc

# Program Listing for File Console.cc¶

↰ Return to documentation for file (`Console.cc`)

```
/*--------------------------------------------------------------------------*\
 |                                                                          |
 |  Copyright (C) 2020                                                      |
 |                                                                          |
 |         , __                 , __                                        |
 |        /|/  \               /|/  \                                       |
 |         | __/ _   ,_         | __/ _   ,_                                |
 |         |   \|/  /  |  |   | |   \|/  /  |  |   |                        |
 |         |(__/|__/   |_/ \_/|/|(__/|__/   |_/ \_/|/                       |
 |                           /|                   /|                        |
 |                           \|                   \|                        |
 |                                                                          |
 |      Enrico Bertolazzi                                                   |
 |      Dipartimento di Ingegneria Industriale                              |
 |      Universita` degli Studi di Trento                                   |
 |      email: enrico.bertolazzi@unitn.it                                   |
 |                                                                          |
\*--------------------------------------------------------------------------*/


#ifndef DOXYGEN_SHOULD_SKIP_THIS

#include "Utils.hh"

#ifdef __clang__
#pragma clang diagnostic ignored "-Wpoison-system-directories"
#pragma clang diagnostic ignored "-Wc++98-compat"
#endif

namespace Utils {

  #ifndef DOXYGEN_SHOULD_SKIP_THIS
  using std::string;
  using std::lock_guard;
  using std::mutex;
  #endif

  void
  Console::change_level( int new_level ) {
    UTILS_ASSERT(
      new_level >= -1 && new_level <= 4,
      "Console::change_level( new_level = {})\n"
      "new_level must be in the range [-1,4]\n",
      new_level
    );
    m_level = new_level;
  }

  void
  Console::change_stream( ostream_type * new_p_stream ) {
    m_stream = new_p_stream;
  }

  Console const &
  Console::black( string const & msg, int msg_level ) const {
    lock_guard<mutex> lock_access(m_message_mutex);
    if ( msg_level <= m_level )
      (*m_stream) << rang::fg::black << msg << rang::fg::reset;
    return *this;
  }

  Console const &
  Console::red( string const & msg, int msg_level ) const {
    lock_guard<mutex> lock_access(m_message_mutex);
    if ( msg_level <= m_level )
      (*m_stream) << rang::fg::red << msg << rang::fg::reset;
    return *this;
  }

  Console const &
  Console::green( string const & msg, int msg_level ) const {
    lock_guard<mutex> lock_access(m_message_mutex);
    if ( msg_level <= m_level )
      (*m_stream) << rang::fg::green << msg << rang::fg::reset;
    return *this;
  }

  Console const &
  Console::yellow( string const & msg, int msg_level ) const {
    lock_guard<mutex> lock_access(m_message_mutex);
    if ( msg_level <= m_level )
      (*m_stream) << rang::fg::yellow << msg << rang::fg::reset;
    return *this;
  }

  Console const &
  Console::blue( string const & msg, int msg_level ) const {
    lock_guard<mutex> lock_access(m_message_mutex);
    if ( msg_level <= m_level )
      (*m_stream) << rang::fg::blue << msg << rang::fg::reset;
    return *this;
  }

  Console const &
  Console::magenta( string const & msg, int msg_level ) const {
    lock_guard<mutex> lock_access(m_message_mutex);
    if ( msg_level <= m_level )
      (*m_stream) << rang::fg::magenta << msg << rang::fg::reset;
    return *this;
  }

  Console const &
  Console::cyan( string const & msg, int msg_level ) const {
    lock_guard<mutex> lock_access(m_message_mutex);
    if ( msg_level <= m_level )
      (*m_stream) << rang::fg::cyan << msg << rang::fg::reset;
    return *this;
  }

  Console const &
  Console::gray( string const & msg, int msg_level ) const {
    lock_guard<mutex> lock_access(m_message_mutex);
    if ( msg_level <= m_level )
      (*m_stream) << rang::fg::gray << msg << rang::fg::reset;
    return *this;
  }

  Console const &
  Console::black_reversed( string const & msg, int msg_level ) const {
    lock_guard<mutex> lock_access(m_message_mutex);
    if ( msg_level <= m_level )
      (*m_stream)
        << rang::fg::black << rang::style::reversed
        << msg << rang::style::reset << rang::fg::reset;
    return *this;
  }

  Console const &
  Console::red_reversed( string const & msg, int msg_level ) const {
    lock_guard<mutex> lock_access(m_message_mutex);
    if ( msg_level <= m_level )
      (*m_stream)
        << rang::fg::red << rang::style::reversed
        << msg << rang::style::reset << rang::fg::reset;
    return *this;
  }

  Console const &
  Console::green_reversed( string const & msg, int msg_level ) const {
    lock_guard<mutex> lock_access(m_message_mutex);
    if ( msg_level <= m_level )
      (*m_stream)
        << rang::fg::green << rang::style::reversed
        << msg << rang::style::reset << rang::fg::reset;
    return *this;
  }

  Console const &
  Console::yellow_reversed( string const & msg, int msg_level ) const {
    lock_guard<mutex> lock_access(m_message_mutex);
    if ( msg_level <= m_level )
      (*m_stream)
        << rang::fg::yellow << rang::style::reversed
        << msg << rang::style::reset << rang::fg::reset;
    return *this;
  }

  Console const &
  Console::blue_reversed( string const & msg, int msg_level ) const {
    lock_guard<mutex> lock_access(m_message_mutex);
    if ( msg_level <= m_level )
      (*m_stream)
        << rang::fg::blue << rang::style::reversed
        << msg << rang::style::reset << rang::fg::reset;
    return *this;
  }

  Console const &
  Console::magenta_reversed( string const & msg, int msg_level ) const {
    lock_guard<mutex> lock_access(m_message_mutex);
    if ( msg_level <= m_level )
      (*m_stream)
        << rang::fg::magenta << rang::style::reversed
        << msg << rang::style::reset << rang::fg::reset;
    return *this;
  }

  Console const &
  Console::cyan_reversed( string const & msg, int msg_level ) const {
    lock_guard<mutex> lock_access(m_message_mutex);
    if ( msg_level <= m_level )
      (*m_stream)
        << rang::fg::cyan << rang::style::reversed
        << msg << rang::style::reset << rang::fg::reset;
    return *this;
  }

  Console const &
  Console::gray_reversed( string const & msg, int msg_level ) const {
    lock_guard<mutex> lock_access(m_message_mutex);
    if ( msg_level <= m_level )
      (*m_stream)
        << rang::fg::gray << rang::style::reversed
        << msg << rang::style::reset << rang::fg::reset;
    return *this;
  }

  Console::Console( ostream_type * stream, int level )
  : m_stream(stream)
  , m_level(level)
  {
    m_message_style.s = rang::style::reset;
    m_message_style.f = rang::fg::reset;
    m_message_style.b = rang::bg::reset;

    m_warning_style.s = rang::style::reset;
    m_warning_style.f = rang::fg::yellow;
    m_warning_style.b = rang::bg::reset;

    m_error_style.s = rang::style::italic;
    m_error_style.f = rang::fg::red;
    m_error_style.b = rang::bg::reset;

    m_fatal_style.s = rang::style::underline;
    m_fatal_style.f = rang::fg::red;
    m_fatal_style.b = rang::bg::reset;
  }

  Console const &
  Console::semaphore(
    unsigned       rvg,
    string const & msg,
    int            msg_level
  ) const {
    lock_guard<mutex> lock_access(m_message_mutex);
    static rang::fg rvg_color[3] = {
      rang::fg::red, rang::fg::yellow, rang::fg::green
    };
    if ( msg_level <= m_level )
      (*m_stream)
        << rang::style::reset
        << rang::bg::reset
        << rvg_color[rvg%3]
        << msg
        << rang::fg::reset;
    return *this;
  }

  Console const &
  Console::message( string const & msg, int msg_level ) const {
    lock_guard<mutex> lock_access(m_message_mutex);
    if ( msg_level <= m_level )
      (*m_stream)
        << m_message_style.s
        << m_message_style.f
        << m_message_style.b
        << msg
        << rang::style::reset
        << rang::fg::reset
        << rang::bg::reset;
    return *this;
  }

  Console const &
  Console::warning( string const & msg ) const {
    lock_guard<mutex> lock_access(m_message_mutex);
    if ( m_level >= 2 )
      (*m_stream)
        << m_warning_style.s
        << m_warning_style.f
        << m_warning_style.b
        << msg
        << rang::style::reset
        << rang::fg::reset
        << rang::bg::reset;
    return *this;
  }

  Console const &
  Console::error( string const & msg ) const {
    lock_guard<mutex> lock_access(m_message_mutex);
    if ( m_level >= 1 )
      (*m_stream)
        << m_error_style.s
        << m_error_style.f
        << m_error_style.b
        << msg
        << rang::style::reset
        << rang::fg::reset
        << rang::bg::reset;
    return *this;
  }

  Console const &
  Console::fatal( string const & msg ) const {
    lock_guard<mutex> lock_access(m_message_mutex);
    (*m_stream)
      << m_fatal_style.s
      << m_fatal_style.f
      << m_fatal_style.b
      << msg
      << rang::style::reset
      << rang::fg::reset
      << rang::bg::reset;
    return *this;
  }

}

#endif
```

### Quick search

### Table of Contents

- rang - Colors for your Terminal
- Zstr
- C++ API

«
hide menu

menu
sidebar
»

### Navigation

- index
- toc
- Utils »
- Program Listing for File Console.cc

© Copyright 2021, Enrico Bertolazzi.
Created using Sphinx 4.2.0.
